# Meta-Analysis of the Clinical Value of Danshen Injection and Huangqi Injection in Liver Cirrhosis

**DOI:** 10.1155/2013/842824

**Published:** 2013-08-28

**Authors:** Changtai Zhu, Hao Cao, Xifa Zhou, Chunlei Dong, Judong Luo, Changsong Zhang, Jinming Liu, Yang Ling

**Affiliations:** ^1^Department of Radiation Oncology, Changzhou Tumor Hospital Soochow University, Changzhou 213000, China; ^2^Department of Laboratory Medicine, Changzhou Tumor Hospital Soochow University, Changzhou 213000, China; ^3^Department of Cardiothoracic Surgery, Shanghai East Hospital, Tongji University School of Medicine, Shanghai, China; ^4^Department of Oncology, Changzhou Tumor Hospital Soochow University, Changzhou 213000, China; ^5^Department of Respiratory Medicine, Shanghai Pulmonary Hospital, Tongji University School of Medicine, Shanghai, China

## Abstract

*Objective*. To evaluate the clinical value of Danshen injection and Huangqi injection for the treatment of liver cirrhosis. *Methods*. The Chinese Biomedical Literature Database (CBM), Chinese Scientific Journals Full-Text Database (VIP), Wanfang Database, China National Knowledge Infrastructure (CNKI), PubMed, and EMBASE database were searched to collect the literatures about the randomized controlled trials involving the treatment of liver cirrhosis with Danshen injection combined with Huangqi injection, and the data analyses were performed using RevMan 4.2 software. *Results*. A total of 11 studies involving 1086 patients (trials group: 554 cases, control group: 532 cases) were included in this study. Compared with those in control group, the meta-analysis showed-that the total effectiveness rate and the level of serum albumin increased, while serum total bilirubin, alanine transmninase, type III procollagen, hyaluronic acid, laminin, and type-IV collagen decreased in trials group. The Jadad score ranged from 1 to 2 and the funnel plot analysis suggests that publication bias may occur. *Conclusions*. Danshen injection combined with Huangqi injection may promote the curative efficacy of liver cirrhosis, which is a promising novel treatment approach. The exact outcome needs to perform rigorously designed, multicenter, and large randomized controlled trials.

## 1. Introduction

Liver fibrosis or cirrhosis is a common progressively pathological lesion of chronic liver diseases in response to various liver-damaging factors [1]. The main causes of liver fibrosis in industrialized countries include chronic HCV infection, alcohol abuse, and nonalcoholic steatohepatitis [2]. In China, cirrhosis is most commonly caused by hepatitis B and hepatitis C, because China has the largest number of hepatitis patients in the world. Of the 350 million to 400 million individuals worldwide infected with the hepatitis B virus (HBV), one-third reside in China, with 130 million carriers and 30 million chronically infected [3, 4]. Presently, the treatment for liver cirrhosis remained difficult, due to the lack of effective approaches. In recent years, many clinical trials using Danshen injection and Huangqi injection for the treatment of liver cirrhosis have been reported in China, suggesting that the new therapeutic approach may have potential value. However, there lacked a systematical review about the issue. Therefore, we conducted a meta-analysis of RCTs to assess the clinical value of Danshen injection and Huangqi injection for the treatment of liver cirrhosis.

## 2. Materials and Methods

### 2.1. Inclusion Criteria

The studies were randomized controlled trials (RCTs) and quasi-randomized controlled trials. Clinical diagnosis must meet the diagnostic criteria for cirrhosis of the liver (Chinese Commission of Infectious and Parasitic Diseases, Viral Hepatitis Prevention and Treatment Programs). The trials group added Danshen or compound Danshen injection and Huangqi injection apart from the drugs that were used by the control group. The outcome should include one or more indices as follows: (1) total efficacy rate; (2) serum indices of liver function: alanine aminotransferase (ALT), total bilirubin (TBIL), and albumin (ALB); (3) serum indices of liver fibrosis: hyaluronic acid (HA), type-IV collagen (VI-C), type-III procollagen (PC-III) and laminin (LN); (4) adverse events; E other data related to efficacy and outcome.

In this study, we had not set any restrictions on gender, race, and literature language.

### 2.2. Exclusion Criteria

Reviews, nonclinical studies, case observations, duplicated literatures, and noninjection formulae literatures were excluded.

### 2.3. Research Strategy and Data Extraction

**Figure 1:**
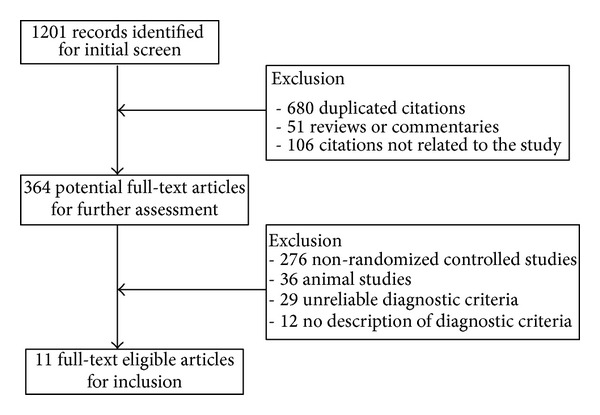
Flow diagram of the literature selection for this study.

Danshen, dan shen, huangqi, Beiqi, huang qi, salvia, salvia miltiorrhiza, astragalus, astragali, astragalus miltiorrhiza, Chinese traditional medicine herb, fibrosis, cirrhosis of liver, cirrhosis, liver cirrhosis, and liver fibrosis were selected as the search terms. The Chinese Biomedical Literature Database (CBM), China National Knowledge Infrastructure (CNKI), Chinese Scientific Journals Full-text Database (VIP), Wanfang Database, PubMed, and EMBASE database were searched by computer. Data extraction and quality assessment was independently performed by two researchers (C. Zhu and Y. Ling) and disagreements were resolved by consensus. The lack of information was supplemented by contact with the authors in charge of the clinical trials. Database retrieval process is shown in Figure 1. The methodological quality of trials was assessed by Jadad score. The randomization, concealment of allocation, blind methods, and loss of followup were considered as the scoring evidence.

### 2.4. Statistical Analysis

Statistical analysis was performed by Cochrane RevMan 4.2. Categorical variables were compared using relative risk (RR), and continuous variables were compared using weighted mean difference (WMD). Meanwhile, 95% confidence interval (CI) was calculated. Chi-square test was used for the heterogeneity of inclusion trials. The heterogeneity data adopted the random effect model was used, otherwise fixed effect model. A funnel plot was used for assessing the potential publication bias.

## 3. Results

### 3.1. Characteristics of Included Studies

Eleven articles [5–15] involving 1086 subjects (trials group: 554 cases; control group: 532 cases) were included in this study. Male patients with liver cirrhosis were dominated in the subjects. The general characteristics of the study were shown in Table 1, and interventions, treatments and outcomes were seen in Table 2.

### 3.2. The Quality Assessment

The design features clarified as randomization, double-blinding, withdrawals/dropouts, and allocation concealment were shown in Table 3. The Jadad scores ranged from 1 to 2. In this study, the curative effect assessment was performed by the clinicians and double-blinding design was not adopted. But, the measurements of serum indices of liver function and liver fibrosis were independently conducted by laboratory staff, and the double-blinding method was performed in all the included studies. However, the withdrawals/dropouts and allocation concealment were not conducted. 

### 3.3. Meta-Analysis of Assessing the Total Effectiveness Rate

**Figure 2:**
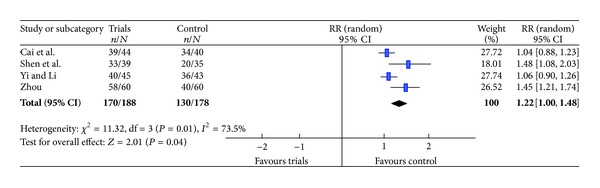
Meta-analysis of assessing the total efficacy rate.

Four RCTs [5, 7, 8, 11] have evaluated the total efficacy rate. Heterogeneity analysis of the total efficacy rate was significant difference (*P* < 0.05), and a random effects model was used. The results showed the total efficacy rate in the trials group was higher than that in the control group (RR = 1.22, 95% CI (1.0, 1.48); *P* < 0.05) (Figure 2). 

### 3.4. Meta-Analysis of the Serum Indices of Liver Function

**Figure 3:**
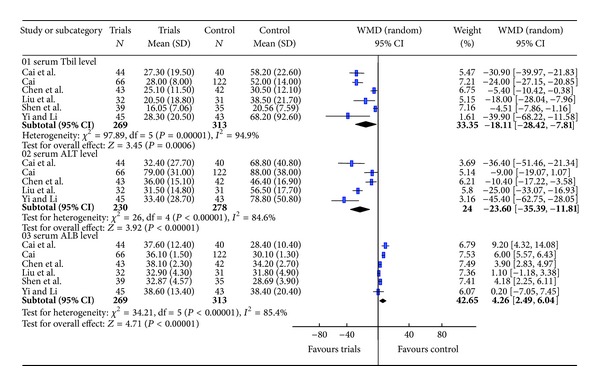
Meta-analysis of the serum indices of liver function (TBIL, ALT and ALB).

The heterogeneity occurred in the data of ALT, TBIL, and ALB (*P* < 0.05); therefore, we adopted a random effects model. The results showed that the levels of ALT and TBIL in the trials group decreased significantly compared with the control group, while the ALB level increased. The WMDs with 95% CI of ALT, TBIL and ALB were −18.11 (−28.42, −7.81), −23.60 (−35.39, −11.81) and 4.26 (2.49, 6.04), respectively. Forest plots were shown in Figure 3.

### 3.5. Meta-Analysis of the Serum Indices of Liver Fibrosis

**Figure 4:**
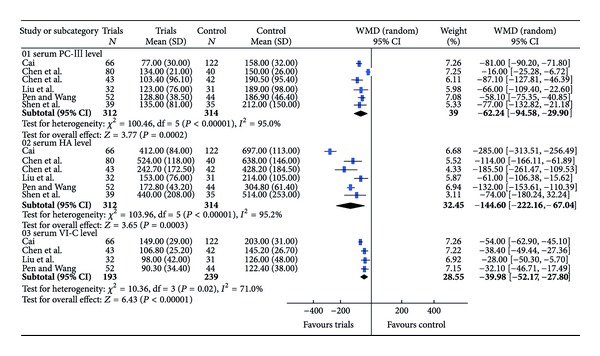
Meta-analysis of the serum indices of liver fibrosis (PCIII, HA and CIV).

**Figure 5:**
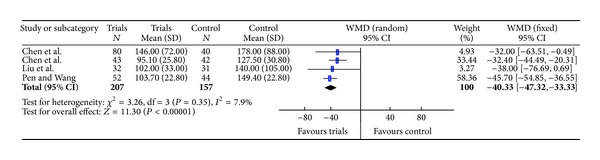
Meta-analysis of the indices of liver fibrosis (LN).

Heterogeneity analyses implied that PC-III, HA, and VI-C fit random effects model, while LN should adopt a fixed effects model. Meta-analysis showed that the WMDs of PC-III, HA, LN, and VI-C with 95% CI were −62.24 (−94.58, −29.90), −144.60 (−222.16, −67.04), −40.33 (−47.32, −33.33), and −39.98 (−52.17, −27.80), respectively, suggesting that the levels of serum PCIII, HA, LN, and VI-C in the trials group were lower than those in control group (Figures 4 and 5).

### 3.6. Liver Hemodynamics, HBeAg Seroconversion and HBV-DNA

Wu et al. [12] reported hemodynamic changes in liver as follows: after 4 weeks of treatment, portal vein diameter, the inner diameter, and blood flow velocity of the splenic vein in the trials group significantly decreased, while the flow velocity of portal vein blood significantly increased (*P* < .05). Liu et al [15] reported that no difference in the HBeAg seroconversion rate was found between the trials group and the control group (*P* > .05), while the copies of HBV-DNA in the trials group were significantly lower than those in the control group (*P* < .05).

### 3.7. Recurrence Rate

Cai et al. [7] reported that, when 3 months after treatment, 2 cases recurred in the trials group (38 cases received followup); while in the control group (20 cases received followup), 6 cases recurred. There was statistical difference (*P* < .05) between the two groups.

### 3.8. Adverse Effect

Amongst 11 literatures included in this study, only one [12] reported side effects and clarified that no side effects in clinical trials were observed.

### 3.9. Publication Bias

**Figure 6:**
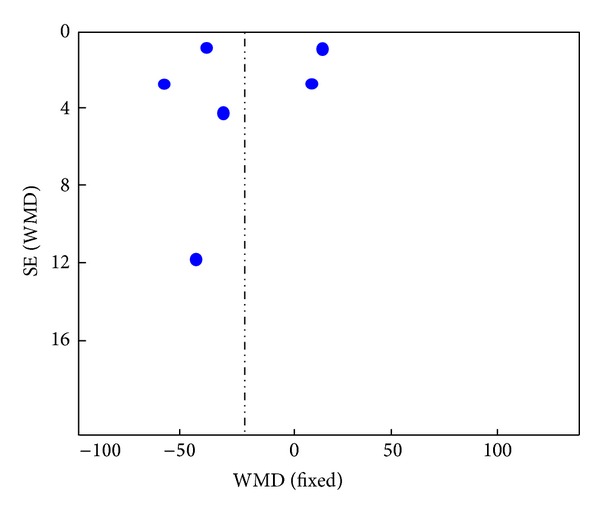
The funnel plot based on the data of TBIL.

According to the data involving TBIL, the funnel plot was drawn using WMD as abscissa and SE (WMD) for the vertical axis, respectively. The plot was asymmetric (Figure 6), suggesting that the publication biases may occur in this study.

## 4. Discussion

Liver fibrosis results from chronic damage to the liver in conjunction with the accumulation of ECM proteins including collagen, which is a characteristic of most types of chronic liver diseases [16]. In contrast with the traditional view that cirrhosis is an irreversible disease, recent evidence indicates that even advanced fibrosis is reversible [17, 18]. However, up to now, there has been no standard Western medicine treatment for liver fibrosis. In traditional Chinese medicine (TCM), some Chinese herbs such as *Radix Salviae Miltiorrhizae* and *Radix Astragali Mongolici* were commonly used in cases of liver fibrosis and hepatitis. In recent years, the extract of their roots using ethanol was prepared as Danshen injection and Huangqi injection, respectively, which were often prescribed as the treatment for liver fibrosis. However, in China, Danshen injection was commonly used for coronary heart disease [19], myocarditis [20], stroke disease [21], myocardial infarction [22], hepatitis [23], epidemic hemorrhagic fever [24], acute disseminated intravascular coagulation syndrome so on [25]. Huangqi injection has been widely used in the treatment for chronic hepatitis [26], viral myocarditis [27], chronic heart failure [28], aplastic anemia [29], chronic nephritis [30], renal damage [31], nephrotic syndrome [32, 33], and diabetic nephropathy [34]. 

*Radix Salviae Miltiorrhizae* contains tanshinones, salvianolic acids, rosmarinic acid, lithospermic acid, protocatechualdehyde, caffeic acid, isoferulic acid, and so forth. It has been demonstrated that the ingredients can inhibit the proliferation and activation of stellate cells, and promote the repair and regeneration of liver cells, improve microcirculation in the liver, and increase hepatic parenchymal blood supply [35, 36]. Modern pharmacological studies have shown that, *Radix Astragali Mongolici* contains a series of cycloartane triterpene glycosides denoted astragalosides I–VII (saponins), which can inhibit the synthesis and the deposition of collagen, and promote the protein synthesis and the recovery of the cell function in liver [37–39].

In this study, Danshen injection and Huangqi injection combined with routine therapeutic regiment (the control) were used for the clinical treatment of cirrhosis. The treatment protocols were as follows: Danshen injection (10–30 mL) and Huangqi injection (10–30 mL) were respectively or together added into 250–500 mL of 5%–10% glucose solution for intravenous drip, qd, and the course of treatment ranges from 28 d to 90 d. Meta-analysis showed that the therapeutic effect in the trials group (using Danshen injection and Huangqi injection) was better than that in the control group (routine treatment). Serum ALT, ALB, and TBIL are the main markers of evaluating liver function, and the meta-analysis implied that using Danshen injection and Huangqi injection can decrease the serum levels of ALT, ALB, and TBIL, suggesting that the injections improve recovery of liver function. HA, LN, VI-C, and PC-III are the serum markers of the therapeutic evaluation of liver fibrosis; meta-analysis results showed that; the serum levels of HA, LN, VI-C, and PC-III in the trials group were lower compared with those in the control group. Thus, the treatment was believed to relieve liver fibrosis. In addition, individual study [12] showed that, after 4 weeks of treatment using Danshen injection and Huangqi injection, portal vein diameter, the inner diameter, and blood flow velocity of the splenic vein decreased, while the flow velocity of portal vein blood increased, indicating that the liver thermodynamics were bettered after the treatment using the injections. In addition, Liu et al. [15] reported that the copies of HBV-DNA had significantly reduced after the treatment.

Based on the above results, we believed that using Danshen injection and Huangqi injection combined with the routine treatment may improve liver function recovery and reverse liver fibrosis. But, it must be noted that this meta-analysis had some limitations. Firstly, the quality of the methodological design of individual studies was not high. Secondly, the asymmetric funnel plot implied that publication biases may occur. Thirdly, the diversity of treatment dose and the small sample number and the lack of long-term follow-ups degraded the validity of the evidence of the clinical trials. In addition, the safety of the trials was reported insufficiently, and there only had one literature that mentioned side effect and none else clarified the safety observation. However, the previous study [40] reported that, using Huangqi injection in treating chronic hepatitis B patients showed flustered symptoms, and the symptoms disappeared after slowing drip rate. In addition, another study reported that the rarely moderate anaphylaxis in intravenous dripping of Danshen injection may occur [41].

## 5. Conclusions

Danshen injection combined with Huangqi injection may promote the curative efficacy of liver cirrhosis, which is a promising novel treatment approach. Considering that this systematic review had the limitations in some ways, rigorously designed multicenter, double-blind, randomized, and large-scale controlled trials are required.

## Figures and Tables

**Table 1:** Characteristics of the randomized controlled trials included in this study.

Author (reference)	Published year	Cases T/C	Age (years) Range, mean	Sex Male/female	History (years) Range, mean
Shen et al. [5]	1999	39/35	T: 39–72, 55.3 C: 40–71, 56.5	T: 26/13 C: 23/12	2–13, 5.7
Chen et al. [6]	2000	80/40	NR	NR	NR
Cai et al. [7]	2001	44/40	T: 8–51, 29 C: 12–50, 31	T: 35/9 C: 32/8	T: 0.8–11, 5.9 C: 0.7–11, 5.8
Zhou [8]	2002	60/60	T: 28–70, 49 C: 26–68, 47	T: 40/20 C: 45/15	NR
Liu et al. [9]	2002	56/44	35–67	78/2	3–25
Chen et al. [10]	2003	43/42	36–71, 54.72	NR	NR
Cai [11]	2004	66/122	T: 54.6 C: 58.7	T: 59/7 C: 107/15	NR
Wu et al. [12]	2006	37/31	35–65	51/17	>2
Yi and Li [13]	2007	45/43	T: 20–55, 37 C: 18–51, 33	T: 25/20 C: 25/18	T: 5–13 C: 5–10
Pen and Wang [14]	2010	52/44	T: 28–69, 36.8 C: 24–67, 35.6	T: 38/14 C: 28/16	NR
Liu et al. [15]	2010	32/31	25–62, 39.7	T: 26/6 C: 23/8	NR

T: trials group, C: control group. NR: no report.

**Table 2:** The intervention and the outcome measures of the individual studies included in this study.

Study ID	The regimens of intervention		Time	Assessment of outcomes
	Trials group	Control group		
Shen et al., 1999 [5]	Compound Danshen injection (16 mL) and Huangqi injection (20 mL) were, respectively, added into 5%–10% glucose solution (250 mL), intravenously, qd.	Aspartic potassium, vitamin C, and B6 were added into 5% glucose, intravenously.	28 d	Total efficacy rate; serum indices of liver fibrosis and liver function.
Chen et al., 2000 [6]	Compound Danshen injection (20 mL) and Huangqi injection (20 mL) were, respectively, added into 5% glucose solution, intravenously, qd.	Used hepatoprotective treatment (mainly energy mixture and vitamins), qd.	90 d	Serum indices of liver fibrosis.
Cai et al., 2001 [7]	Compound Danshen injection (20 mL) and Huangqi injection (20 mL) were, respectively, added into 5% glucose solution, intravenously, qd.	Used silymarin orally, tid.	90 d	Total efficacy rate; serum indices of liver function.
Zhou, 2002 [8]	Compound Danshen injection (20 mL) and Huangqi injection (10 mL) were together added into 250 mL of 5% glucose solution, intravenously, qd.	Vitamin C, vitamin B6, inosine, and potassium chloride injection were added into 5% glucose solution intravenously, qd.	75 d	Total efficacy rate; serum indices of liver function.
Liu et al., 2002 [9]	Danshen injection (30 mL) and Huangqi injection (20 mL) were, respectively, added into 250 mL of 5% glucose solution, intravenously, qd.	Added ganlixin into the 5% glucose, intravenously.	90 d	Serum indices of liver function.
Chen et al., 2003 [10]	Danshen injection (30 mL) and Huangqi injection (16 mL) were, respectively, added into 250 mL of 5% glucose solution, intravenously, qd.	30 mL of ganlixin injection, intravenously, qd.	28 d	Serum indices of liver fibrosis and liver function.
Cai, 2004 [11]	Compound Danshen injection (30 mL) and Huangqi injection (10 mL) were, respectively, added into 250 mL of 5% glucose solution, intravenously, qd.	Energy mixture, branched-chain amino acids, albumin, and vitamin were used.	75 d	Serum indices of liver fibrosis and liver function.
Wu et al., 2006 [12]	Danshen injection (25 mL) and Huangqi injection (25 mL) were, respectively, added into 250 mL of 5% glucose solution, intravenously, qd.	Routine western medicine treatment.	84 d	Serum indices of liver function; liver hemodynamics.
Yi and Li, 2007 [13]	Danshen injection (20 mL) and Huangqi injection (20 mL) were, respectively, added into 250 mL of 5% glucose solution, intravenously, qd.	Used silymarin orally, 3 times a day.	90 d	Total efficacy rate; serum indices of liver function.
Pen and Wang, 2010 [14]	Danshen injection (30 mL) and Huangqi injection (30 mL) were, respectively, added into 250 mL of 5% glucose solution, intravenously, qd.	Hepatoprotective therapy using nosine and vitamins.	30 d	Serum indices of liver fibrosis and liver function.
Liu et al., 2010 [15]	Danshen injection (30 mL) and Huangqi injection (30 mL) were, respectively, added into 250 mL of 10% glucose solution, intravenously, qd.	Hepatoprotective and supportive therapy and lamivudine (100 mg) orally, qd.	90 d	Serum indices of liver fibrosis and liver function; HBeAg; HBV-DNA.

The regimens in the trials group included the drugs used in the control group. Compound Danshen injection: mainly contains Danshen and an added constituent (*Lignum Dalbergiae Odoriferae*).

**Table 3:** Quality of reports of 11 clinical trials using the Jadad assessment scale.

	Randomization	Double blinding	Withdrawals/dropouts	Allocation concealment	Scores
Curative effect assessment	Yes	No	No	No	1
Serum indices of liver function	Yes	Yes	No	No	2
Serum indices of liver fibrosis	Yes	Yes	No	No	2
